# Assessing Regional and Interspecific Variation in Threshold Responses of Forest Breeding Birds through Broad Scale Analyses

**DOI:** 10.1371/journal.pone.0055996

**Published:** 2013-02-07

**Authors:** Yntze van der Hoek, Rosalind Renfrew, Lisa L. Manne

**Affiliations:** 1 Department of Biological Sciences, College of Staten Island and Graduate Center, City University of New York, New York City, New York, United States of America; 2 Vermont Center for Ecostudies, White River Junction, Vermont, United States of America; 3 Department of Biological Sciences, College of Staten Island, City University of New York, New York City, New York, United States of America; Stockholm University, Sweden

## Abstract

**Background:**

Identifying persistence and extinction thresholds in species-habitat relationships is a major focal point of ecological research and conservation. However, one major concern regarding the incorporation of threshold analyses in conservation is the lack of knowledge on the generality and transferability of results across species and regions. We present a multi-region, multi-species approach of modeling threshold responses, which we use to investigate whether threshold effects are similar across species and regions.

**Methodology/Principal Findings:**

We modeled local persistence and extinction dynamics of 25 forest-associated breeding birds based on detection/non-detection data, which were derived from repeated breeding bird atlases for the state of Vermont. We did not find threshold responses to be particularly well-supported, with 9 species supporting extinction thresholds and 5 supporting persistence thresholds. This contrasts with a previous study based on breeding bird atlas data from adjacent New York State, which showed that most species support persistence and extinction threshold models (15 and 22 of 25 study species respectively). In addition, species that supported a threshold model in both states had associated average threshold estimates of 61.41% (SE = 6.11, persistence) and 66.45% (SE = 9.15, extinction) in New York, compared to 51.08% (SE = 10.60, persistence) and 73.67% (SE = 5.70, extinction) in Vermont. Across species, thresholds were found at 19.45–87.96% forest cover for persistence and 50.82–91.02% for extinction dynamics.

**Conclusions/Significance:**

Through an approach that allows for broad-scale comparisons of threshold responses, we show that species vary in their threshold responses with regard to habitat amount, and that differences between even nearby regions can be pronounced. We present both ecological and methodological factors that may contribute to the different model results, but propose that regardless of the reasons behind these differences, our results merit a warning that threshold values cannot simply be transferred across regions or interpreted as clear-cut targets for ecosystem management and conservation.

## Introduction

Motivated largely by indications of declining wildlife populations due to habitat loss and fragmentation [1], [2], [3] ecologists established a vast body of work on species-habitat relationships over the last decades. From these studies, it became apparent that wildlife responses to habitat loss and fragmentation are often non-linear [4], [5], [6], [7], [8]. An increasing number of studies support the main prediction of the extinction threshold hypothesis that there are certain critical amounts of habitat required for population persistence [9]. These thresholds are often defined as a ‘range of habitat cover below which the probability of population persistence decreases dramatically’ [10], [11]. Documentation of thresholds’ existence led to a rise of interest in determining critical habitat amount or minimum patch size for species or population persistence [1], [12], [13], [14], [15]. More recently, threshold modeling extended to incorporate landscape-scale thresholds, leading to reasonable evidence for thresholds in relationships between species occurrence and habitat cover at landscape extents [16], [17], [18]. In addition, first attempts in determining habitat thresholds in persistence dynamics over time, rather than mere occurrence at a single point in time, have been fruitful and comprise a useful contribution to landscape ecology and conservation alike [19].

Although determination of persistence and extinction thresholds are now considered major focal points of research [20], [21], [22], [23], uncertainty and debate on numerous issues continue to persist. These issues include the mechanisms that are driving threshold responses [24], [25], confounding factors [26], [27] and the value of applying threshold modeling approaches in conservation planning and management [28], [29], [30], [31]. One of the main concerns regarding the incorporation of threshold analyses in conservation is the lack of generality and transferability of results across species [32], [33], [34] and regions [18], [35].

Spatial and interspecific variation in critical thresholds has been widely recognized [18], [19], [36], [37], but few studies have directly aimed at quantifying the mechanisms driving variation (but see [18], [38], [39]). In order to derive conservation targets and generalizations regarding species-habitat relationships, we will need to study spatial and interspecific variation more intensely [35], [40], [41] and conduct studies across more species and larger geographic areas than previously pursued. In order to do so, we might be required to step away from time and budget-limited smaller scale field studies [18], [42] and instead focus on existing data sources for broad-scale analyses. Zuckerberg and Porter [19] provide an example of a methodology through which we can assess threshold responses of breeding birds on a broad (state-wide) scale. For their study they make use of breeding bird atlas data. These data provide a unique opportunity to model species distributions and species-habitat relationships, because of the broad scale and number of species for which analyses can be made [19], [43], [44], [45], [46]. In addition, many regions (states, countries) have repeated breeding bird atlas projects, usually spaced 20–25 years apart, thus allowing us to compare between regions and to address spatial variation in long-term persistence rather than occurrence at a single point in time [19].

Here, we present a first multi-region comparison in a larger study that quantifies variation in thresholds found in long-term species-habitat relationships at a landscape scale. We conducted an analysis of threshold responses across a large set of breeding forest birds at the scale of an entire state (Vermont) in a similar fashion to the aforementioned study by Zuckerberg and Porter [19]. Subsequently, we compared their results to ours in what is to our knowledge the first attempt of a regional comparison of threshold responses. Here, we highlight the potential that the use of state-wide breeding bird atlases has for threshold analyses and how our approach may answer existing questions regarding the generality and transferability of models and results [18], [35], [47], [48]. In addition, we propose that our approach holds potential for further investigation of traits that may be correlated with species-specific area-sensitivity [33], [40], [41] and mechanisms that drive geographic variation in thresholds responses [18]. In this paper, we do not go into depth on the reasons behind regional variation in threshold responses, but do highlight potential ecological explanations as well as methodological biases that may lead to difference in model outcomes. Finally, we investigate how scale influences threshold responses by comparing models that include habitat cover at different scales. Due to the grid-based design of our data set (i.e. atlas blocks) these kinds of comparisons across scales are straightforward, thus affording a unique opportunity to address the influence of scale on species-habitat relationships [32], [46], [49], [50].

## Methods

Bird distribution atlases present a unique opportunity to study occurrence dynamics on a broad scale and over a relatively long term [19]. We based our analyses on a repeated atlas project, The Atlas of Breeding Birds of Vermont, for which 1976–1981 [51] and 2003–2007 [52] data were available. We thereafter compared our results with a previous analysis by Zuckerberg and Porter [19] that is based on data from the 1980–1985 and the 2000–2005 New York State Breeding Bird Atlases. Surveys for these atlases were conducted following a largely similar protocol, allowing analyses without large sampling biases. We will hereby briefly summarize the sampling approach that was used for the Vermont atlases, as fully described in Renfrew [52], whereas further details on the New York State Atlases can be found in McGowan and Corwin [53] and Zuckerberg and Porter [19].

**Figure 1:**
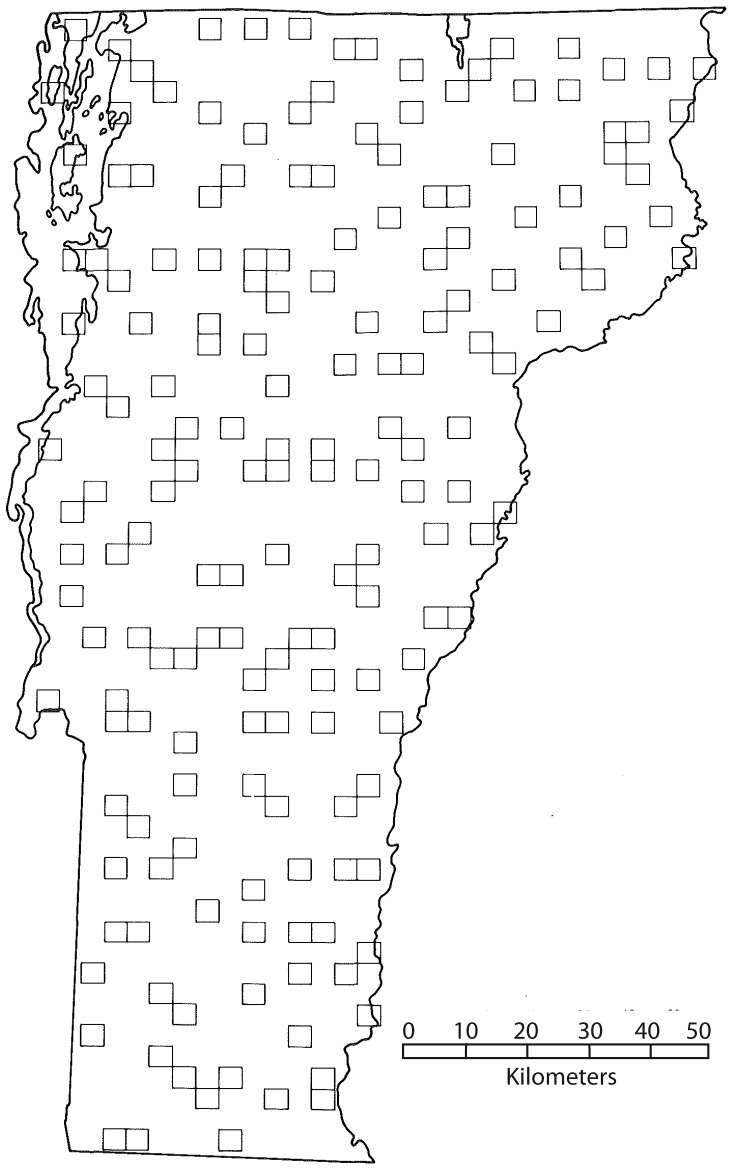
Map of priority blocks of the First and Second Vermont Breeding Bird Atlas Projects.

Both Vermont Breeding Bird Atlas Projects were based on systematic sampling of predetermined blocks by both volunteer and expert fieldworkers. These fieldworkers were expected to determine both species occurrence and breeding bird status in each of the surveyed blocks. Blocks measured approximately 25 km^2^, and were based on a grid derived from U.S. Geological Survey maps. Due to limited manpower, 179 randomly selected priority blocks were assigned for which adequate coverage was to be achieved (Figure 1). The overall aim for the Vermont Breeding Bird Atlas Projects was to determine breeding bird occurrence at three different levels of confidence (‘possible breeding’, ‘probable breeding’, and ‘confirmed breeding’) [51].

For our purpose, and to keep consistency with the protocol followed by Zuckerberg and Porter [19], we considered a bird present when it was listed in an atlas under any of the aforementioned levels of confidence. In order to investigate how this decision with regards to the inclusion of data influences our results we also provide a sensitivity analysis in which we only consider a bird present in the case of ‘confirmed breeding’.

The Vermont Breeding Bird Atlas Project considered blocks to be satisfactorily surveyed when 75 or more species were observed, while at least 35 had to be confirmed as breeders. This corresponded to observations of roughly 75% of the total number of species likely to occur in an average atlas block, and nesting confirmation for half of these species [51]. Each block was surveyed by volunteers during the breeding season until, over the course of a 1–5 year period, the aforementioned predetermined coverage standard was reached [51].

The second atlas followed a similar approach [52] to the protocol outlined in the first atlas. In our analyses, we excluded data from four atlas blocks because they were not fully located within Vermont, and thus did not overlap exactly with our Vermont land cover data, or because the majority of the block consisted of water.

We assessed the change in occurrence from the first to the second atlas as persistence or extinction for each focal species. As in Zuckerberg and Porter [19] we classified birds as persistent in an atlas block when found in both the first and second atlas, and extinct as detected in the first but not the second atlas. Focal species were 25 forest generalists or obligates, and were the same as those studied by Zuckerberg and Porter [19]. This selection did not include rare species in order to avoid issues of low sample size and detection biases.

We determined the percentage of forest cover in each atlas block using 1992 National Land Cover Data (NLCD) (for an accuracy analysis see [54]). Zuckerberg and Porter [19] used this same data set for their analysis in New York. The date of the land cover data is midway between the two atlas projects, and we assumed that loss or gain of forest in Vermont between 1978 (onset, first atlas) and 2007 (last sampling year, second atlas) was small and thus not biasing. We based this assumption on the small change in forest cover in the Northeastern Highlands Ecoregion, which includes Vermont, between 1973 and 2000 (from 85.2% to 81.4% of the total landcover) [55]. The HISTO command in the software program IDRISI Taiga [56] provided a numeric frequency histogram from which the percentage of forest cover for each atlas block could be calculated. We combined the percentages of the land cover types “mixed”, “deciduous” and “coniferous” forest to account for the total forest cover in each block.

**Figure 2:**
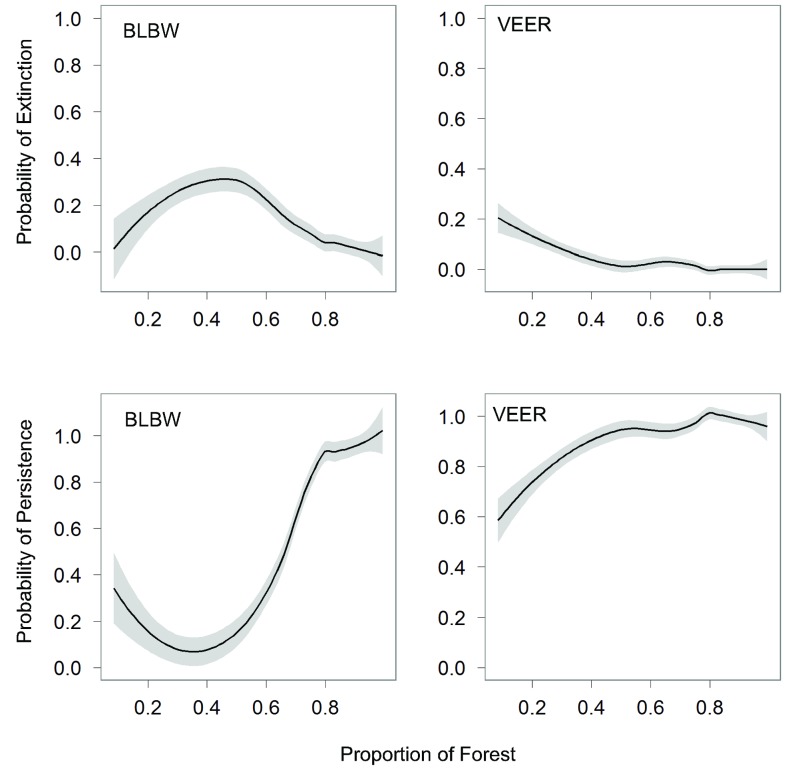
Loess plots of persistence and extinction dynamics for two bird species. Loess plots showing the relationship between percentage of forest cover in an atlas block and the probability of persistence or extinction for two species (BLBW = Blackburnian Warbler (*Dendroica fusca*); VEER = Veery (*Catharus fuscescens*)). These plots were used for visual assistance in finding initial values for our segmented regression algorithms and visual checks only.

**Figure 3:**
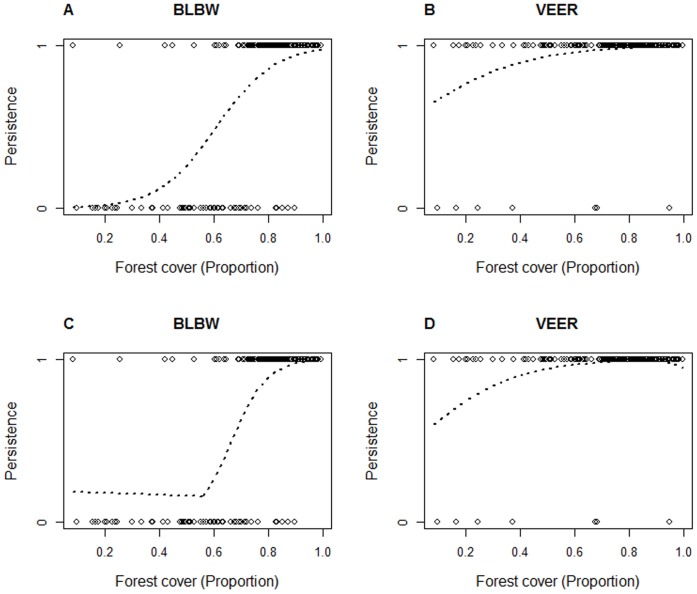
Plots of persistence data and logistic (Panel A–B) and segmented (Panel C–D) regression models. Plots showing point data (1 = persisting, 0 = not persisting) and either a fitted logistic or segmented regression model for two species (BLBW = Blackburnian Warbler (*Dendroica fusca*); VEER = Veery (*Catharus fuscescens*)). These plots were used to provide a visual check of the actual data and fitted models (in addition to the loess plots). The plots for Blackburnian Warbler are illustrative for a species with strong support for a threshold model, whereas both the data distribution and model plots of Veery are indicative of low support for a threshold model (additional figures for all species can be found in Figure S1). In the case of Veery, this might simply be due to it being persistent in the majority of atlas blocks.

We built threshold (segmented logistic regression [57]) and non-threshold (logistic regression) models to describe the relationship between forest cover and occupancy dynamics using R [57]. We used the fitted values of locally weighted nonparametric models (loess plots) with a smoothing parameter of 0.75 to visualize empirical relationships between forest cover (%) and occurrence and to identify initial values for segmented regression models (Figure 2). We explored all possible initial values between 0 and 100% forest cover in 5% steps (e.g. 35%, 40%) when model algorithms failed to converge using our initial starting point [18], [19]. We conducted our statistical analyses in R [58], using standard packages and the ‘segmented’ package [59]. In total, we fitted 50 logistic models (25 species×2 dynamics) and 50 segmented logistic regression models in this study (Figure 3). We took an information-theoretic approach on selecting the best models describing species-habitat amount relationships using Akaike’s Information Criterion (AIC) and delta AIC (Δ_i_). We selected a model as best model if the alternative model had Δ_i_ >2 [60]. Both models could be considered equivalent in their support when Δ_i_ <2 between threshold and non-threshold models [60]. In this case, we selected the model with the least parameters: the non-threshold model. Subsequently, we determined the fit of the best models to the original data by calculating the Area Under the Curve (AUC) [60] using the ROCR package [61]. In general, AUC values vary between 0 and 1, with 1 representing a perfect fit and 0.5 representing that the model fits no better than a random prediction would. Models with AUC values >0.7 are usually considered acceptable, while values >0.8 are considered excellent [18], [62].

Sample size might have pronounced effects on the power to detect thresholds and the percentage of forest cover at which the thresholds are estimated [63] and could thus potentially explain (part of) differences in results the Vermont (N = 175 atlas blocks) and New York (N = 5074 atlas blocks) analyses. We investigated the effect of sample size by obtaining 5000 random subsets of 175 blocks from the entire New York data set and fitted a segmented regression and logistic regression to each of these subsets. We repeated this procedure for all 25 species and both dynamics (persistence and extinction). Based on the aforementioned selection criteria (AIC, delta AIC) we obtained information on the number of times (out of 5000 simulations) threshold models were selected as the better model and the distribution and range of the associated breakpoints (measured in percentage of forest cover).

Although spatial autocorrelation is considered an important aspect of analyses such as ours [32], [64], [65], the atlas blocks used in our analysis were sufficiently spaced apart to exclude this as a source of error (Figure 1). This sample design allowed us to investigate the relationship between persistence, or extinction, and habitat amount on a larger scale. We repeated earlier outlined models of species responses, using forest cover (%) in an atlas block and all the surrounding eight atlas blocks as an independent variable. We compared AIC scores and threshold estimates of these new models with our original models and determined whether forest cover in the wider landscape or merely forest cover in the atlas block was a better predictor of species persistence or extinction.

## Results

Logistic and segmented regression models converged for most species and provided us with a base for model selection (Table 1, Table S1). However, for Red-eyed Vireo (*Vireo olivaceus*) none of the models converged because it was persistent in all atlas blocks. In addition, no threshold models converged for Black-capped Chickadee (*Poecile atricapilla*) and Hermit Trush (*Catharus guttatus*) with regards to persistence, which is likely due to this species being persistent in all but few atlas blocks (Figure S1).

**Table 1:** Comparisons between logistic (non-threshold) and segmented (threshold) regression models for four forest breeding birds, with the best models highlighted in bold.

Species	Dynamic	Model	AIC	Δ_i_	%^1^	SE	AUC
Yellow-bellied Sapsucker(*Sphyrapicus varius*)	Persistence	**Threshold**	**113.21**	**0**	**34.61**	**10.97**	**0.84**
		Non-Threshold	116.10	2.89			
	Extinction	Threshold	31.92	0	42.65	142.60	
		**Non-Threshold**	**33.65**	**1.73**			**0.65**
Blue-headed Vireo (*Vireo solitarius*)	Persistence	Threshold	165.47	0	91.23	8.59	
		**Non-Threshold** 2	**169.80**	**4.33**			**0.82**
	Extinction	**Threshold**	**77.17**	**0**	**50.82**	**5.03**	**0.70**
		Non-Threshold	80.64	3.47			
Black-and-white Warbler(*Mniotilta varia*)	Persistence	**Threshold**	**75.77**	**0**	**87.96**	**5.83**	**0.82**
		Non-Threshold	79.52	3.75			
	Extinction	**Threshold**	**39.70**	**0**	**91.20**	**5.67**	**0.78**
		Non-Threshold	41.90	2.20			
Yellow-rumped Warbler(*Dendroica coronata*)	Persistence	**Threshold**	**151.47**	**0**	**19.45**	**3.83**	**0.80**
		Non-Threshold	161.00	9.53			
	Extinction	**Threshold**	**112.03**	**0**	**78.67**	**3.76**	**0.73**
		Non-Threshold	118.50	6.47			

1 Percentage of forest cover associated with the response threshold.

2 Although the AIC and AUC values indicate support for the threshold model, visual inspection of the loess plot did not support the existence of a threshold.

For extinction dynamics, we found that 9 out of 25 supported models included a threshold parameter (alternative non-threshold model Δ_i_ >2, Table S1). Six other species showed support for a threshold model (with regard to both AIC and AUC values), but we deemed the standard errors for the breakpoint estimates too large to consider these valid models to incorporate in further analyses. For persistence dynamics, threshold models were selected to describe the habitat-relationships of 5 species (Table S1). Two more species, Winter Wren (*Troglodytes troglodytes*) and Blue-headed Vireo (*Vireo solitaries*), would have supported threshold models based on mere AIC values, but visual interpretation of plotted data (Figure S1) did not support the existence of any thresholds and thus we did not include these species in further analyses. We interpret our results as moderate support for threshold response at landscape scale, in contrast to Zuckerberg and Porter [19] who used a similar approach and the same set of focal species and found overall strong support for threshold models. They concluded that 15 species showed support for the inclusion of a threshold parameter in extinction responses in New York, and no less than 21 of 25 species supported threshold models for persistence [19].

Estimates of threshold forest amounts in extinction responses varied considerably between species, ranging from 50.82% (SE = 5.03) for the Blue-headed Vireo (*Vireo solitaries*) to 91.02% (SE = 5.67) for the Black-and-white Warbler (*Mniotilta varia*) (Table 1, Table S1). For persistence, threshold estimates varied from 19.45% (SE = 3.83) for the Yellow-rumped Warbler (*Dendroica coronata*) to 87.96% (SE = 5.83) for the Black-and-white Warbler. Discriminatory power of all selected models was high, with an average AUC of 0.73 (SE = 0.02) for non-threshold models and 0.76 (SE = 0.03) for threshold models. Although threshold models were in general less often selected in our analysis than in Zuckerberg and Porter’s [19], we did find that the selected models provided a good fit with 11 out of 14 threshold models having an AUC value >0.7 and 8 out of 14 models having an AUC >0.75 (Table S1).

**Figure 4:**
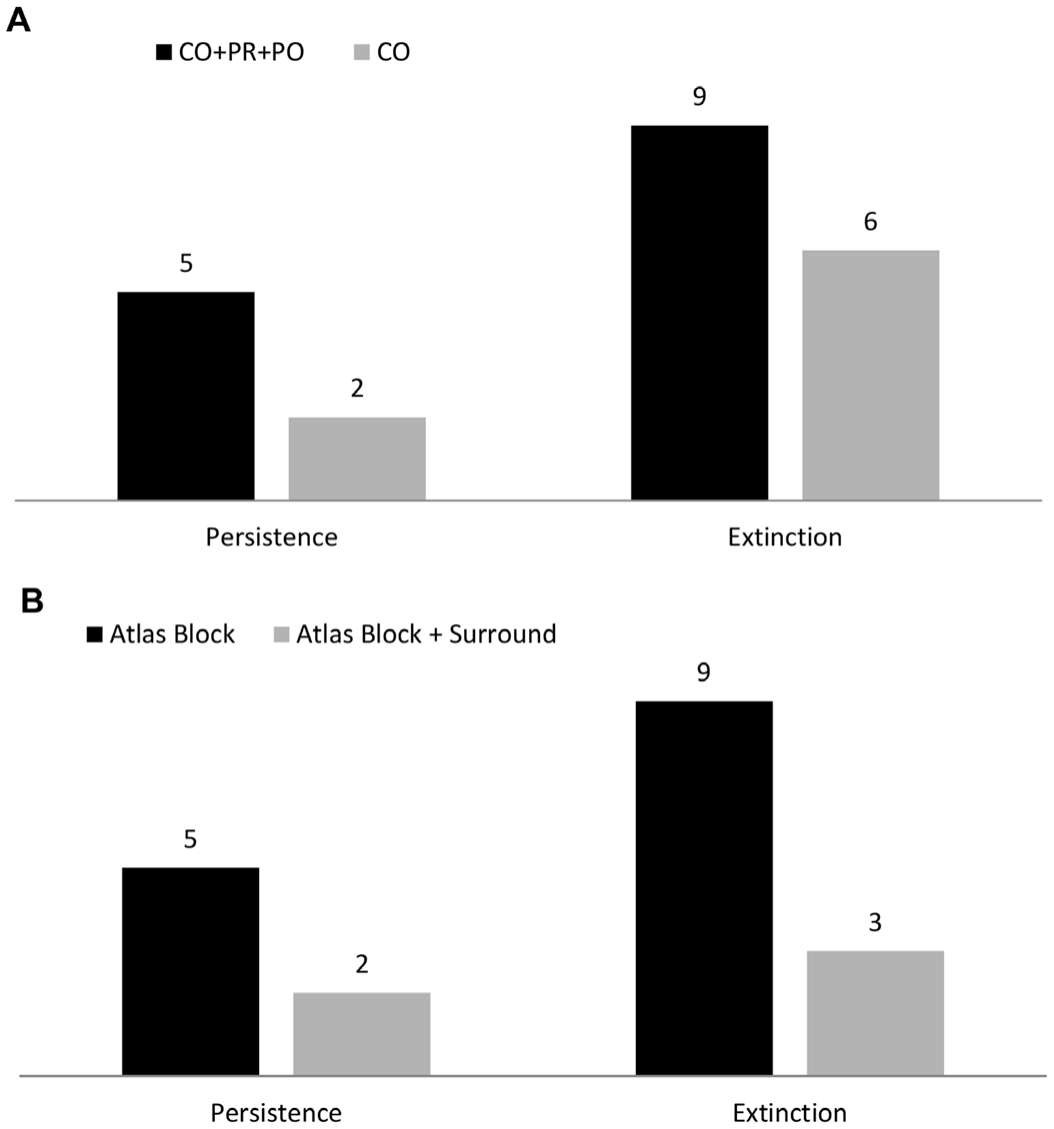
Mean persistence and extinction threshold estimates for New York and Vermont. Depicted are the means of estimated thresholds in forest cover below which the probability of persistence or extinction declined rapidly. We calculated the means across all estimated thresholds of breeding bird species that showed support for threshold models in both states (only 5 species for persistence and 9 for extinction). Error bars show the associated standard error. *The mean of the threshold estimates for New York State was calculated based on thresholds reported by Zuckerberg and Porter [19].

Not only did the support for threshold responses in Vermont differ from that of New York [19], but the estimated threshold values for species also differed between the two states (Table 2). Four out of five species that showed support for persistence thresholds in both states had lower associated threshold estimates (threshold at lower proportion of forest cover) in Vermont as compared to New York. On average, the 5 species that supported a threshold model for persistence in both states had an associated threshold estimate of 61.41% (SE = 6.11) in New York and 51.08% (SE = 10.60) in Vermont (Figure 4). Seven species showed threshold responses in extinction in both states, with average threshold values of 66.45% (SE = 9.15) in New York and 73.67% (SE = 5.70) in Vermont (Figure 4).

**Table 2:** Comparison of estimated threshold values (% of forest cover) between New York and Vermont.

		New York^1^		Vermont	
Species	Dynamic	%^2^	SE	%^2^	SE
Blue-headed Vireo (*Vireo solitarius*)	Extinction	85.62 (60)	4.82	50.82	5.03
Black-and-white Warbler (*Mniotilta varia*)	Persistence	39.79 (36)	5.72	87.96	5.83
	Extinction	39.90 (66)	6.85	91.20	5.67
Magnolia Warbler *(Dendroica magnolia*)	Extinction	84.41 (72)	3.68	83.91	4.64
Yellow-rumped Warbler (*Dendroica coronata*)	Persistence	73.61 (44)	4.70	19.45	3.83
Black-throated G. Warbler (*Dendroica virens*)	Extinction	32.31 (58)	3.64	73.82	21.49
Blackburnian Warbler (*Dendroica fusca*)	Persistence	77.35 (75)	3.93	56.26	6.61
	Extinction	82.87 (73)	6.81	69.91	5.26
Canada Warbler (*Wilsonia canadensis*)	Extinction	88.16 (45)	1.99	87.40	3.55
Dark-eyed Junco (*Junco hyemalis (hyemalis)*)	Persistence	59.68 (59)	2.16	57.12	7.43
	Extinction	51.91 (51)	3.73	58.65	5.90
Yellow-bellied Sapsucker (*Sphyrapicus varius*)	Persistence	56.64 (38)	5.30	34.61	10.97

1 We derived threshold values for New York state from Zuckerberg and Porter [19]. In parentheses: The percentage of forest cover at which we found the maximum kernel density for threshold estimates of all selected (out of 5000) subsamples in our simulation approach. These subsamples consisted of 175 atlas blocks randomly taken from the original New York data.

2 Percentage of forest cover associated with the response threshold.

**Figure 5:**
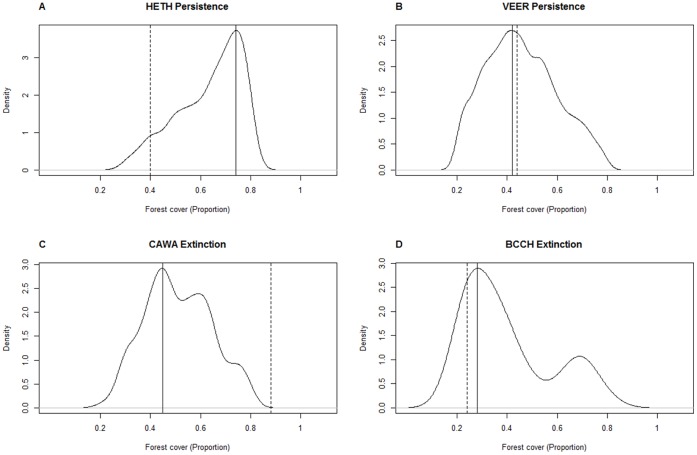
Kernel density plots of estimated breakpoints for all subsamples (out of 5000). We superimposed an estimate of the proportion of forest cover (the threshold) associated with the maximum kernel density of our sampling results (vertical full line) and the threshold derived by Zuckerberg and Porter (2010) (vertical dotted line). Panel A shows results for models of persistence for Hermit Trush (*Catharus guttatus*), Panel B persistence for Veery (*Catharus fuscescens*), Panel C extinction for Canada Warbler (*Wilsonia canadensis*) and Panel D extinction of Black-capped Chickadee (*Poecile atricapilla*). Results for the species shown were exemplary for situations where the estimated thresholds seemingly matched with (Veery, Black-capped Chickadee) or deviated from (Hermit Trush, Canada Warbler) the proportion of forest cover associated with maximum kernel density. Similar plots for all species can be found in Figure S2.

Differences in the number of atlas blocks used in the Vermont and New York analyses might be one of the factors contributing to differences in threshold estimates. We obtained threshold models and associated forest cover estimates that were at times very different from the results as presented in Zuckerberg and Porter [19] when we randomly selected 5000 subsamples of 175 atlas blocks (the sample size for Vermont) from the entire New York data and subsequently fitted threshold and non-threshold models (Figure 5, Figure S2). For most species, threshold models were selected as best models for less than half of the subsamples (<2500). More strikingly, the threshold estimates associated with supported threshold models ranged from approximately 5 to 95% forest cover for all species. When we compare the threshold estimate as derived by Zuckerberg and Porter [19] with the distribution of threshold estimates from our simulation of subsets we see that our subsampling approach hardly gave different results for some species (e.g. persistence for Veery) but very different ones for others (e.g. persistence for Hermit Trush) (Figure 5, Figure S2, Table 2).

We included bird presence at all levels of confirmation (‘possible’, ‘probable’ and ‘confirmed’) in our analyses. However, we do note that similar analyses based on only the most conservative data (‘confirmed’) give us considerably different results. Not only did fewer species show support for threshold models for persistence (2 species) and extinction (6 species) dynamics (Table 3, Figure 4), the critical threshold estimates associated with these models also differed from our original analysis. Threshold models for persistence were supported for Blue-headed Vireo (*Vireo solitaries*) (threshold estimate at 94.50% forest cover (SE = 2.72)) and Scarlet Tanager (82.84%, SE = 6.75). Neither of these species supported a threshold model for persistence in our original analysis (including all levels of occurrence). Three species, Magnolia Warbler (*Dendroica magnolia*) (79.63%, SE = 6.29), Yellow-rumped Warbler (*Dendroica coronate*) (98.20%, SE = 8.12) and Blackburnian Warbler (*Dendroica* fusca) (46.43%, SE = 15.21) supported an extinction threshold model in our conservative approach as well as our initial analysis, but with different threshold estimations. Red-breasted Nuthatch (*Sitta canadensis*) (60.24%, SE = 9.58), Winter Wren (*Troglodytes troglodytes*) (97.41%, SE = 16.07) and Veery (*Catharus fucescens*) (31.52%, SE = 9.30) supported threshold models for extinction only in our more conservative (confirmed breeding only) approach.

**Table 3:** Threshold values (% of forest cover) for species that supported a threshold model when only ‘confirmed’ breeding was considered in the analysis.

Species	Dynamic	Model	AIC	%^1^	SE
Red-breasted Nuthatch (*Sitta canadensis*)	Extinction	**Threshold**	**180.6**	**60.24**	**9.58**
		Non-Threshold	192.3		
Winter Wren (*Troglodytes troglodytes*)	Extinction	**Threshold**	**192.8**	**97.41**	**16.07**
		Non-Threshold	195.6		
Veery (*Catharus fuscescens*)	Extinction	**Threshold**	**172.9**	**31.52**	**9.30**
		Non-Threshold	176.4		
Blue-headed Vireo (*Vireo solitarius*)	Persistence	**Threshold**	**97.7**	**94.50**	**2.72**
		Non-Threshold	100.6		
Magnolia Warbler *(Dendroica magnolia*)	Extinction	**Threshold**	**168.5**	**79.63**	**6.29**
		Non-Threshold	174.5		
Yellow-rumped Warbler *(Dendroica coronata*)	Extinction	**Threshold**	**161.8**	**98.20**	**8.12**
		Non-Threshold	168.5		
Blackburnian Warbler (*Dendroica fusca*)	Extinction	**Threshold**	**177.0**	**46.43**	**15.21**
		Non-Threshold	181.5		
Scarlet Tanager (*Piranga olivacea*)	Persistence	**Threshold**	**134.1**	**82.84**	**6.75**
		Non-Threshold	137.4		

1 Percentage of forest cover associated with the response threshold.

In order to analyze the influence of forest amount in the surrounding landscape on both persistence and extinction dynamics, we created a new set of models that included the average forest cover of the focal atlas blocks plus all eight surrounding blocks as an independent variable. The average forest cover in blocks surrounding atlas blocks was not significantly correlated with the forest cover within atlas blocks (Spearman rank correlation; *ρ* = 0.08, P = 0.30). Only 5 times was a threshold model selected for one of the 50 models that included forest in the surrounding blocks as an extra parameter (Table 4; Figure 4). Regarding persistence, Common Raven (*Corvus corax*, 80.36%, SE = 1.89), and Black-throated Green Warbler (*Dendroica virens*, 83.91%, SE = 9.23^−12^) showed support for a threshold model that included habitat amount at this broad spatial extent. Neither species supported a threshold model for persistence in the original analysis that was based on forest cover in the atlas block alone. Brown Creeper (*Certhia americana*, 59.51%, SE = 2.94), Black-throated Green Warble, 66.31%, SE = 17.71) and Blackburnian Warbler (*Dendroica fusca*, 65.56%, SE = 0.84) showed support for a threshold model in extinction. Two of these species, Black-throated Green Warbler and Blackburnian Warbler, also supported a threshold response for extinction in the original analysis but at slightly different estimates (Black-throated Green Warbler 73.82%, SE = 21.49; Blackburnian Warbler 69.91% (SE = 5.26), Table S1).

**Table 4:** Comparison between threshold and non-threshold models that included forest in the surrounding blocks as an extra parameter.

Species	Dynamic	Model	AIC	%^1^	SE
Common Raven (*Corvus corax*)	Persistence	**Threshold**	**204.19**	**80.36**	**1.89**
		Non-Threshold	208.50		
Brown Creeper (*Certhia americana*)	Extinction	**Threshold**	**109.13**	**59.51**	**2.94**
		Non-Threshold	113.40		
Black-throated G. Warbler (*Dendroica viren*s)	Persistence	**Threshold**	**140.85**	**83.91**	**9.23^−12^**
		Non-Threshold	149.50	0	0
	Extinction	**Threshold**	**29.44**	**66.31**	**17.71**
		Non-Threshold	32.43	0	0
Blackburnian Warbler (*Dendroica fusca*)	Extinction	**Threshold**	**46.65**	**65.56**	**8.43**
		Non-Threshold	64.50	0	0

1 Percentage of forest cover associated with the response threshold.

## Discussion

Regional and interspecific variation in species responses to habitat loss and fragmentation has previously been recognized and put forward as a warning against generalizing results of threshold studies across species and regions [18], [19], [35], [36], [47]. We adopted a simple yet efficient approach to threshold estimation as proposed by Zuckerberg and Porter [19] and extended it to a region (Vermont) adjacent to their original study area (New York). Even though these two regions are largely similar in landscape characteristics such as forest cover and composition, faunal species composition and climate and latitudinal aspects, we found striking differences in the results of our threshold modeling approach. We found less support for the inclusion of a threshold parameter in models of both persistence and extinction responses in Vermont compared to New York. In addition, there were differences between the two regions in the estimate of thresholds in forest cover below which the probability of persistence dramatically declined or extinction increased for species that did support a threshold model in both states.

**Figure 6:**
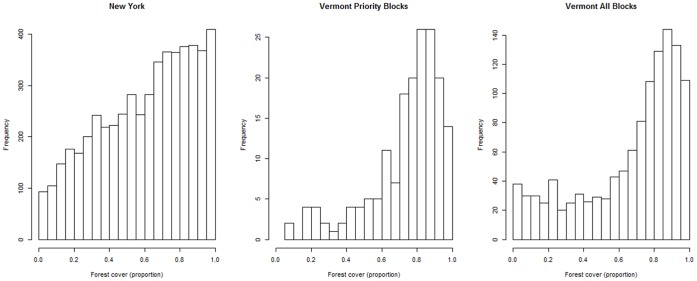
Histograms of forest cover across atlas blocks in the states of Vermont and New York. The frequency of atlas blocks with a particular proportion of forest cover in each of the states. The histograms illustrate that many atlas blocks in both states have high (e.g. more than 0.6, or 60%) levels of forest cover, but also indicate that forest cover across the Vermont atlas blocks is more or less homogeneous (high cover) while atlas blocks in New York have a more variable range of forest cover (i.e. there are many blocks with low forest cover as well). The forest cover of the priority atlas blocks in Vermont seems a representative sample of the forest cover of all atlas blocks in Vermont.

Although the design of the current study did not allow us to assess the cause of regional differences in threshold responses directly, we argue that they might be related to the higher forest cover in Vermont (average atlas blocks 73.89%, SE = 0.02) compared to New York (63.10%, SE = 0.35) (Figure 6). Arguably, high forest cover in the broader surrounding landscape might indicate that forest-associated bird species are able to maintain breeding populations in atlas blocks with low forest cover, because resources (e.g. food resources) can be found in the surrounding atlas blocks with a higher forest cover [17]. In general, atlas blocks with relatively high forest cover are abundant throughout the state of Vermont, whereas a considerable number of atlas blocks with low percentages of forest cover exist in New York (Figure 6).

Another possible explanation for the ability of bird species to apparently persist at lower amounts of forest cover in Vermont as compared to New York, might be related to the ability for bird species to disperse to and from patches within the atlas blocks with lower forest cover. For example, birds might be more likely to be detected as breeding individuals in occasional atlas blocks with low forest cover, due to the existence of source populations in high forest cover atlas blocks in the vicinity. In addition, patches of forest habitat within atlas blocks with low forest cover might be less isolated in Vermont than in New York because surrounding atlas blocks offer patches within dispersal distance [17]. Finally, we might reason that forest composition and structure (forest quality) [16], [35], the quality of the matrix [66], [67] and the level of fragmentation [19], [68] differ between the two study regions and contribute to differences in threshold responses in New York and Vermont. Although fragmentation might play a role in species-habitat relationships it is becoming widely accepted that habitat amount per se is a better predictor of species distributions and responses than fragmentation [10], [27], [46]. Despite this notion, we do argue that incorporating a measure of fragmentation should be part of follow-up studies in order to address whether differences in levels of fragmentation can explain part of the regional differences that appear in threshold estimates [68].

We may have found less support for threshold models in Vermont because the forest cover in most of the surrounding landscape falls above species habitat thresholds [18]. The detection of landscape-scale species-habitat relationships is likely to be lower in regions with high proportions of potential habitat [68]. Pardini et al [69] support this by showing that clear habitat-abundance and habitat-richness effects seem to be lacking in regions with high forest cover. Indeed, most of our study area might have forest cover above the critical values at which thresholds are usually found (10–30%; [4], [17], [20]), obviating the need for a larger study including more study regions.

Species that were considered to follow threshold responses differed in the estimates of threshold values, with persistence thresholds ranging 19.45–87.96% and thresholds in extinction ranging from 50.82% to 91.02% (Table S1). This variation in species-habitat relationships has been widely recognized (e.g. [17], [18], [19], [24]) and been attributed to variation in area-sensitivity as a function of life-history traits such as dispersal capacity [41], [70], reproductive capacity [71], [72] and habitat specialization [69], [72], [73]. Interestingly, in contrast to many previous studies that indicate which species were more area-sensitive than others [18], [19], [36], [69], we did not find consistency in area-sensitivity across regions. For example, when we ranked species according to their threshold estimates for persistence, we found (e.g.) Yellow-rumped Warbler on the high end of the ranking with a threshold value of 73.61% in New York but on the lower end in Vermont (19.45%). The reverse was true for Black-and-white Warbler, having a threshold estimate around 39.79% forest cover in New York but 87.96% in Vermont.

There are many plausible explanations for the results we found and the lack of consistency in threshold values across regions. As mentioned earlier, differences in levels of fragmentation, habitat amount in the wider landscape, quality of the forest and the surrounding matrix are all factors that might be correlated with variation in threshold response. Yet, some of the variation could simply come from problems in defining ‘habitat’ properly. We used a coarse variable (‘forest’) in order to facilitate comparisons with earlier work by Zuckerberg and Porter [19], even though the breeding distribution of many of our focal birds might be better predicted by more specific variables such as ‘evergreen’, ’mixed’ or ‘deciduous’ forest or even subcomponents thereof (particular tree species, prevalence of structural components such as standing dead wood) [18], [74]. Indeed, variation in these more specific characteristics might be large between and within regions, and might thus result in different outcomes in threshold model studies.

Interestingly, thresholds in extinction and persistence did not always seem to occur at the same percentage of forest cover. This difference was most notable in Yellow-rumped Warbler with a threshold in persistence at 19.45% (SE = 3.83) and 78.67% (SE = 3.76) for extinction. Due to an overall low number of species that supported threshold models, we are not able to explore this difference further in our current study, but we argue that differences in thresholds of extinction and persistence might be related to time-lags in regime shifts [69]. Species might seem to persist at percentages of forest cover that are lower than those where peaks in extinction probability occur because of a time-lag in the extinction process. We aim to address this interesting observation further in follow-up studies, in line with arguments that any study that addresses long-term population dynamics should address the phenomenon of time-lags [24].

One important factor in the comparison of thresholds in Vermont and those found in New York is the effect of sample size on our model outcome [63]. The sample size (number of atlas blocks) in Vermont is low (175) compared to New York (5074). We addressed this issue of low sample size by simulating random subsets of 175 atlas blocks from the New York data set and fitting models to these subsets. Interestingly, we found support for a threshold model for less than half of all random subsets and found a wide range of threshold estimates (Figure 5, Figure S1). The latter might be due to the wide range of forest cover found in the atlas blocks in New York (Figure 6), with some of the random subsets containing mainly blocks with low forest cover and others mainly blocks with high forest cover. This is arguably less of a problem in Vermont, where forest cover throughout the atlas blocks seems uniformly high and where the ‘subsample’ of 175 priority atlas blocks seems a good representation of the forest cover in all atlas blocks in Vermont (Figure 6). However, it remains an issue that needs to be addressed. We are aware, and have shown here, that sample size may be one potential reason for the difference in outcome in support for thresholds and in associated forest cover estimates [63]. Therefore, this is an additional factor that we have to take into account when we try to extrapolate from one study or species to the next or when we try to infer conservation and management targets based on studies of threshold responses.

Bird atlas data constitutes different levels of confidence in occurrence (possible, probable, confirmed), and our model results depend on which data we include in our analyses. Considering only the most conservative level of occurrence confirmation (confirmed breeding) in our analysis may arguably improve the accuracy of estimating persistence or extinction thresholds. Basing our analysis on a conservative subset of our data (confirmed only) led to higher threshold estimates in those species that supported a threshold model, indicating that the percentage of forest required for persistence might actually be higher than previously noted here and in other studies. However, we did not extend this analysis here for three reasons. First, we wished to follow a protocol similar to the analysis by Zuckerberg and Porter [19] in order to draw consistent and valid comparisons. Second, we found threshold models to be supported in our ‘conservative scenario’ for only 8 species-dynamic combinations (2 persistence, 6 extinction). Finally, detectability of confirmed breeding occurrences might be quite variable and might limit analyses to only those common species that are easily confirmed as breeders. Nevertheless, we suggest that any study that utilizes breeding bird atlas data should incorporate a consideration of the different results that data inclusion decisions may have. Most importantly however, we suggest that these more conservative estimates of forest requirements once more indicate that we might not be able to directly link the outcome of a threshold estimation model to the narrow conservation goals that are sometimes proposed [23].

Smith et al. [46] noted that forest bird occurrence varies directly with habitat amount in the surrounding landscape regardless of landscape size. We did not test for such a relationship, but did observe a lack of evidence for threshold responses when we included habitat in a larger (∼225 km^2^) focal region in our models. The effects of habitat availability at this larger landscape extent might be diminished by a heterogeneous habitat distribution, given that forest cover in atlas blocks was not significantly correlated with forest cover in the surrounding blocks. Similar effects of declining area-sensitivity with increasing landscape scales have been recorded by Desrochers et al [75].

What we have shown is a) an approach for comparison that can easily be repeated on an unprecedented broad scale and will eventually allow comparison across larger numbers of study areas and species than has been attempted previously, and b) a clear indication that threshold effects and/or amounts are not necessarily supported for the same species in even adjacent areas. If further studies are as promising, our methodology will provide insight into interspecific and regional variation of landscape level, long-term threshold dynamics. Ecosystem managers and conservationists would be able to derive generalities on threshold estimate predictions and gain insight into which characteristics are influencing the minimum habitat requirements for their specific region or focal species of interest. Novelties of our approach include: 1) the ability to address long-term thresholds in persistence and extinction [19] rather than occurrence [18], [36]; and 2) analyzing threshold responses with the use of extensive broad-scale sources of data that are already widely available. This extension of an earlier approach by Zuckerberg and Porter [19] allows for comparisons of threshold models at scales that were previously unobtainable due to costs of field work and time consumption. In addition, we also show that 3) species vary in their threshold responses with regard to habitat amount, and that differences between regions are pronounced. This warrants the advice put forward by many ecologists that threshold values cannot simply be transferred across regions [35] or interpreted as clear-cut targets for ecosystem management and conservation [74]. We also warn that 4) sample size (in our study number of atlas blocks) might have large effects on the outcome of threshold studies (see also [63]) and that this might be one factor driving differences between threshold studies. Finally, our results indicate that 5) thresholds in long-term persistence and extinction dynamics can be found across a wide range of habitat cover or area. Generalizations such as “For most species with large home ranges (such as birds), the threshold may generally be located between 30% and 40% of the habitat… in order to protect the most sensitive species and to deal with uncertainty associated with thresholds, to maintain at least 40% of residual habitats” [23] may oversimplify threshold analyses and may be counter-productive to conservation efforts.

We argue that while searching for thresholds in species-habitat relationships remains a valid goal for ecologists and conservationists, the differences in threshold estimates and response between species and regions are of greatest interest. Analyses that address these differences may contribute to conservation by determining which species are most at risk of being affected by habitat loss. Future research should be directed towards broad-scale comparisons in order to gain insights into consistency of species-habitat relationships, driving factors of both interspecific and regional variation, and general mechanisms underlying species-specific area-sensitivity.

## Supporting Information

Figure S1 **Binomial plots of persistence data and associated estimated logistic regression models.** Plots showing point data (1 = persisting, 0 = not persisting) and a fitted logistic regression model for all species. Panel A contains results for persistence, Panel B for extinction.
(PDF)

Figure S2 **Kernel density plot of estimated breakpoints (proportion of forest cover) for all subsamples (out of 5000) that supported a threshold model.** Superimposed on these plots we see an estimation of the proportion of forest cover (the threshold) associated with the maximum kernel density of our sampling results (vertical full line) and the original threshold derived by Zuckerberg and Porter (2010) (vertical dotted line). Panel A contains results for thresholds in persistence, Panel B for thresholds in extinction.
(PDF)

Table S1 **Comparisons between logistic (non-threshold) and segmented (threshold) regression models for 24 study species.** Red-eyed Vireo (*Vireo olivaceu*s) was also included in the analysis but excluded from this table because none of the models converged. This species thus did not support a logistic or a threshold relationship with forest cover. In fact, it was present in all surveyed atlas blocks. We compared the AIC for all models using delta AIC (Δ_i_), and selected a model when Δ_i_ >2 compared to the other model. We selected the model with the least number of parameters (non-threshold) when the difference between two models was Δ_i_ ≤2. In addition, we present the estimated threshold in the percentage of forest cover (%) and the associated standard error (SE) for the threshold models. For the selected models, we also present the Area Under the Curve statistic (AUC). The best model is highlighted in bold.
(DOCX)
