# Supplementary material for: Assessing Regional and Interspecific Variation in Threshold Responses of Forest Breeding Birds through Broad Scale Analyses
Source: PLoS One. 2013 Feb 7;8(2):e55996. doi: 10.1371/journal.pone.0055996 (PMC3567043; doi:10.1371/journal.pone.0055996)
Supplement: Figure S1 — Binomial plots of persistence data and associated estimated logistic regression models. Plots showing point data (1 = persisting, 0 = not persisting) and a fitted logistic regression model for all species. Panel A contains results for persistence, Panel B for extinction. (PDF) [file pone.0055996.s001.pdf]

Figure S1. Binomial plots of persistence data and associated estimated logistic regression models. Plots showing point data (1 = persisting, 0 = not persisting) and a fitted logistic regression model for all species. Panel A contains results for persistence, Panel B for extinction.

A

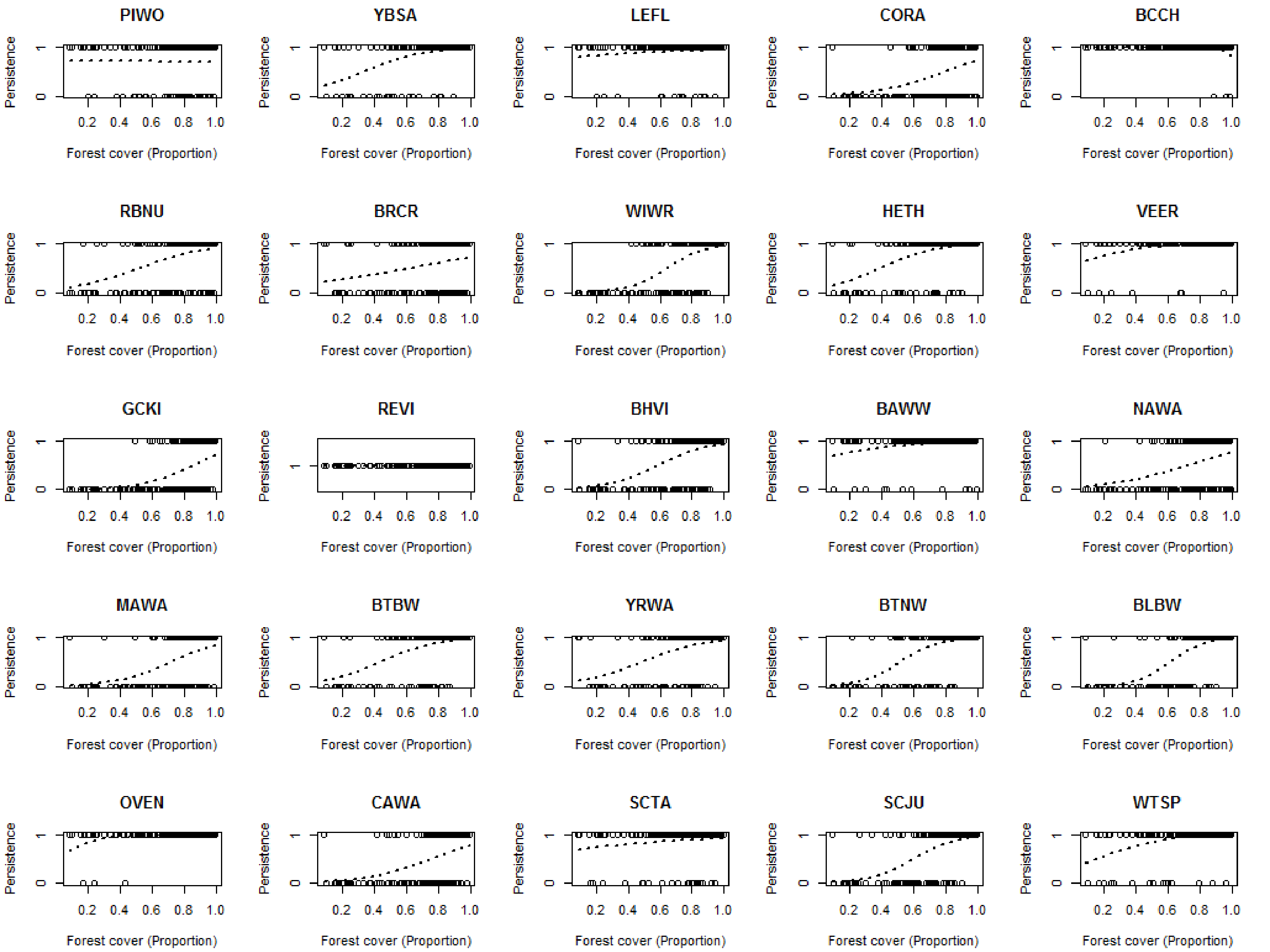

B

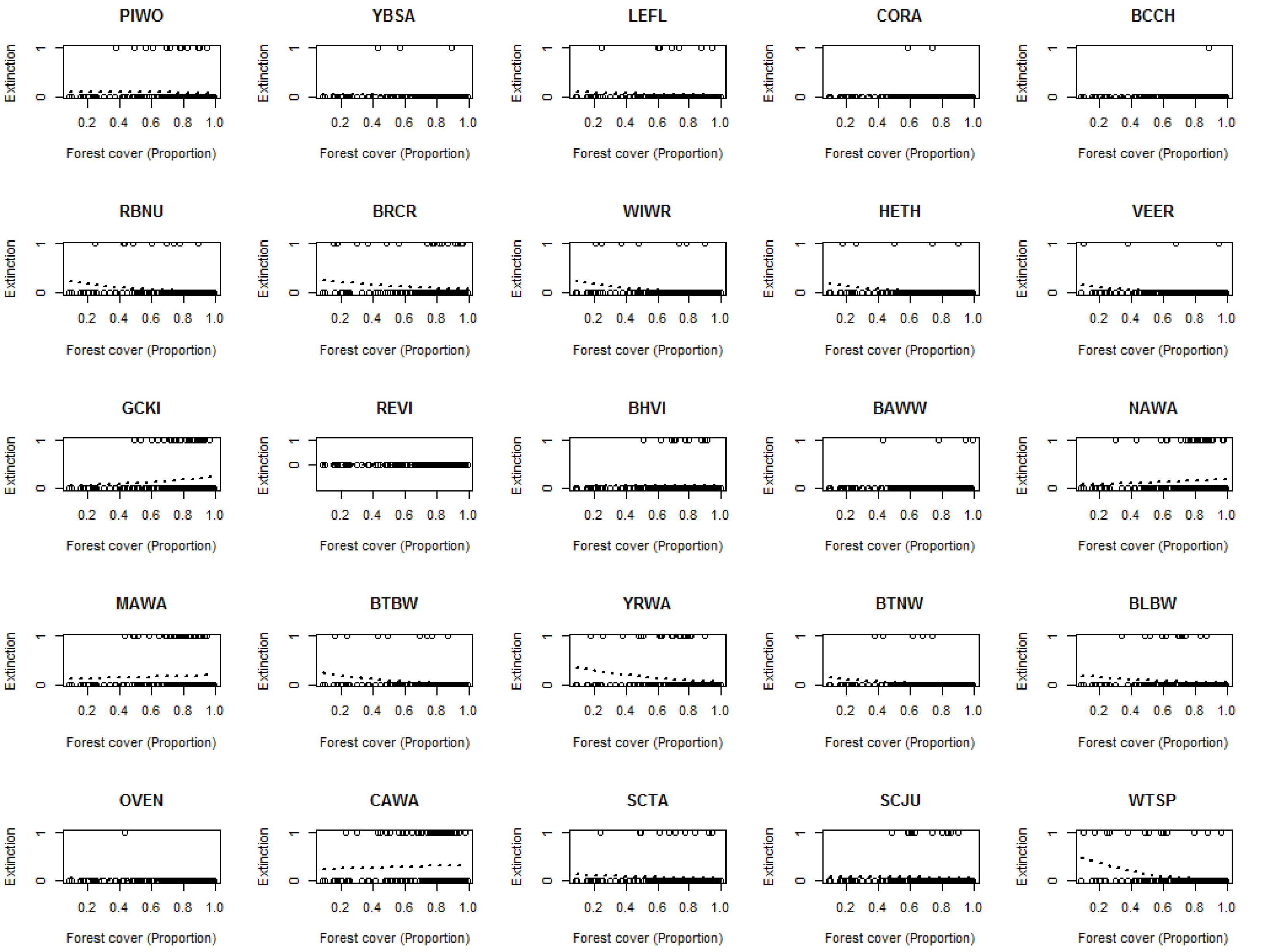

Explanation species codes:

PIWO=Pileated Woodpecker (*Dryocopus pileatus*), YBSA=Yellow-bellied Sapsucker (*Sphyrapicus varius*), LEF=Least Flycatcher (*Empidonax minimus*), CORA=Common Raven(*Corvus corax*), BCCH=Black-capped Chickadee (*Poecile atricapilla*), RBNU=Red-breasted Nuthatch (*Sitta canadensis*), BRCR=Brown Creeper(*Certhia americana*), WIWR=Winter Wren (*Troglodytes troglodytes*), HETH=Hermit Thrush (*Catharus guttatus*), VEER=Veery (*Catharus fuscescens*), GCKI=Golden Crowned-Kingle t(*Regulus satrapa*), REVI=Red-eyed Vireo (*Vireo olivaceus*), BHVI=Blue-headed Vireo (*Vireo solitarius*), BLBW=Black-and-white Warbler (*Mniotilta varia*), NAWA=Nashville Warbler (*Vermivora ruficapilla*), MAWA= Magnolia Warbler (*Dendroica magnolia*), BLBW=Black-throated B. Warbler (*Dendroica caerulescens*), YRWA=Yellow-rumped Warbler (*Dendroica coronata*), BTNW= Black-throated G. Warbler (*Dendroica virens*), BLBW= Blackburnian Warbler (*Dendroica fusca*), OVEN=Ovenbird (*Seiurus aurocapilla*), CAWA=Canada Warbler (*Wilsonia canadensis*), SCTA=Scarlet Tanager (*Piranga olivacea*), Dark-eyed Junco (*Junco hyemalis (hyemalis*)), WTSP=White-throated Sparrow (*Zonotrichia albicollis*)
