# Supplementary material for: Assessing Regional and Interspecific Variation in Threshold Responses of Forest Breeding Birds through Broad Scale Analyses
Source: PLoS One. 2013 Feb 7;8(2):e55996. doi: 10.1371/journal.pone.0055996 (PMC3567043; doi:10.1371/journal.pone.0055996)
Supplement: Figure S2 — Kernel density plot of estimated breakpoints (proportion of forest cover) for all subsamples (out of 5000) that supported a threshold model. Superimposed on these plots we see an estimation of the proportion of forest cover (the threshold) associated with the maximum kernel density of our sampling results (vertical full line) and the original threshold derived by Zuckerberg and Porter (2010) (vertical dotted line). Panel A contains results for thresholds in persistence, Panel B for thresholds in extinction. (PDF) [file pone.0055996.s002.pdf]

Figure S2. Kernel density plot of estimated breakpoints (proportion of forest cover) for all subsamples (out of 5000) that supported a threshold model. Superimposed on these plots we see an estimation of the proportion of forest cover (the threshold) associated with the maximum kernel density of our sampling results (vertical full line) and the original threshold derived by Zuckerberg and Porter (2010) (vertical dotted line). Panel A contains results for thresholds in persistence, Panel B for thresholds in extinction.

A

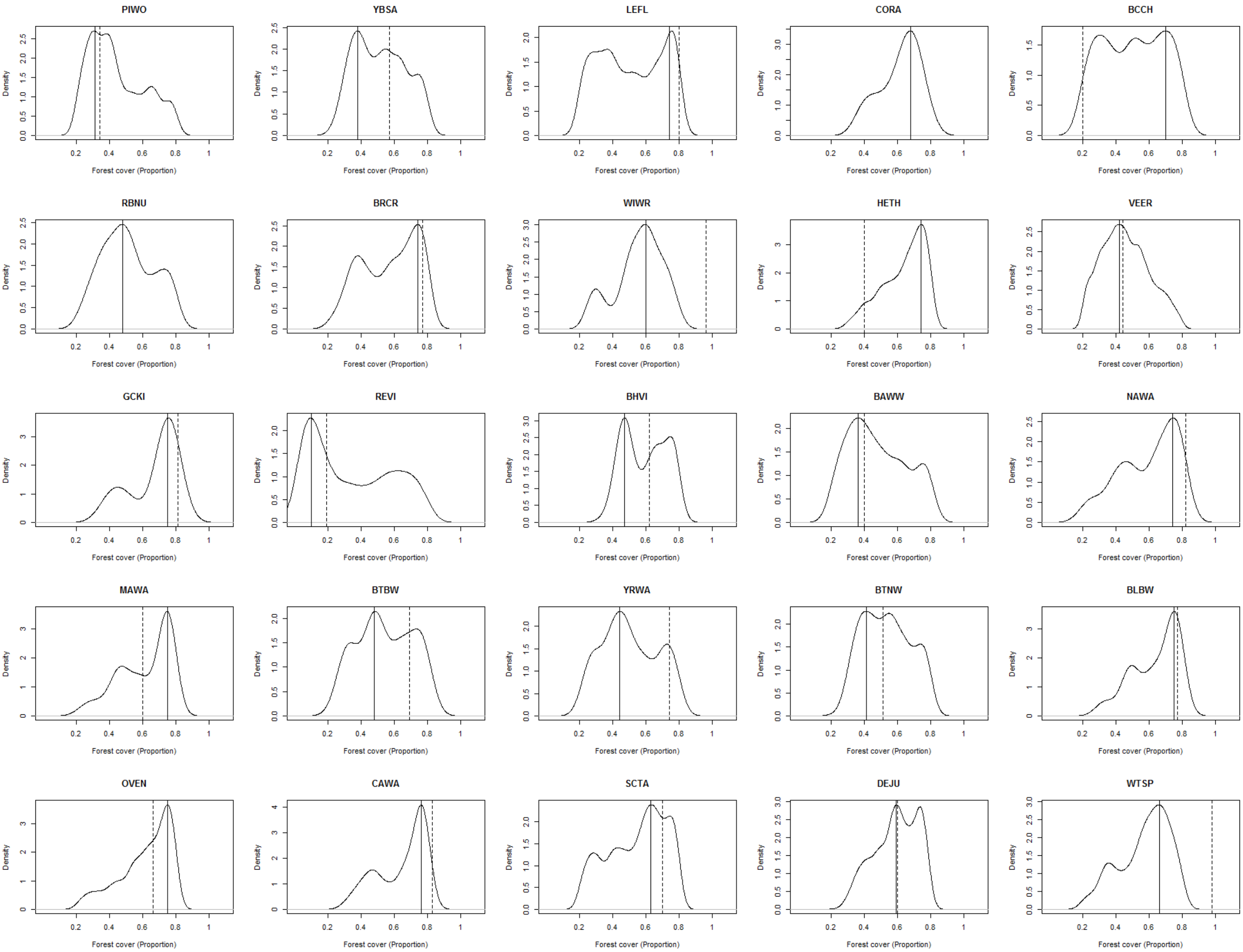

B

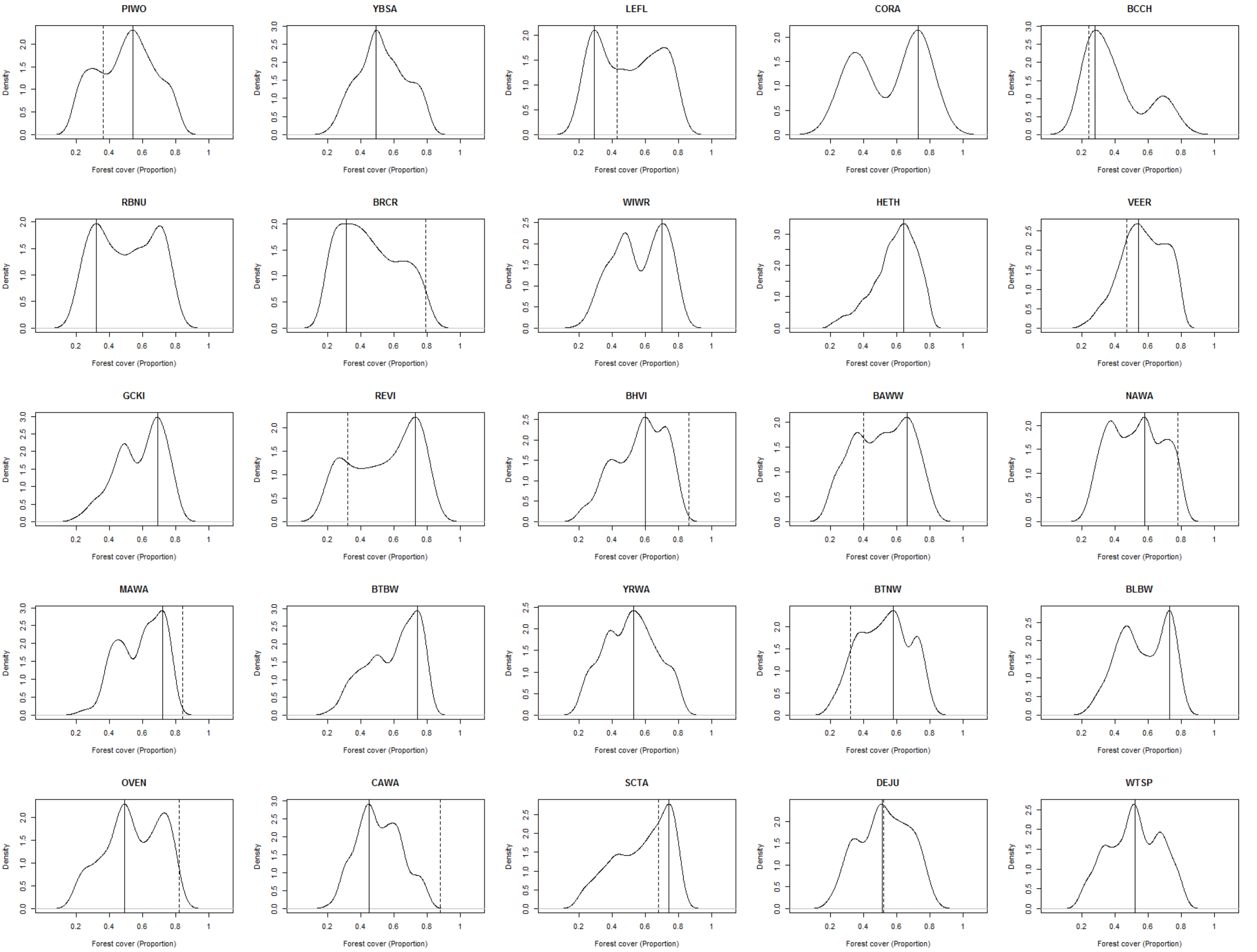

Explanation species codes:

PIWO=Pileated Woodpecker (*Dryocopus pileatus*), YBSA=Yellow-bellied Sapsucker (*Sphyrapicus varius*), LEF=Least Flycatcher (*Empidonax minimus*), CORA=Common Raven(*Corvus corax*), BCCH=Black-capped Chickadee (*Poecile atricapilla*), RBNU=Red-breasted Nuthatch (*Sitta canadensis*), BRCR=Brown Creeper(*Certhia americana*), WIWR=Winter Wren (*Troglodytes troglodytes*), HETH=Hermit Thrush (*Catharus guttatus*), VEER=Veery (*Catharus fuscescens*), GCKI=Golden Crowned-Kingle t(*Regulus satrapa*), REVI=Red-eyed Vireo (*Vireo olivaceus*), BHVI=Blue-headed Vireo (*Vireo solitarius*), BLBW=Black-and-white Warbler (*Mniotilta varia*), NAWA=Nashville Warbler (*Vermivora ruficapilla*), MAWA= Magnolia Warbler (*Dendroica magnolia*), BLBW=Black-throated B. Warbler (*Dendroica caerulescens*), YRWA=Yellow-rumped Warbler (*Dendroica coronata*), BTNW= Black-throated G. Warbler (*Dendroica virens*), BLBW= Blackburnian Warbler (*Dendroica fusca*), OVEN=Ovenbird (*Seiurus aurocapilla*), CAWA=Canada Warbler (*Wilsonia canadensis*), SCTA=Scarlet Tanager (*Piranga olivacea*), Dark-eyed Junco (*Junco hyemalis (hyemalis*)), WTSP=White-throated Sparrow (*Zonotrichia albicollis*)
