# Supplementary material for: Assessing Regional and Interspecific Variation in Threshold Responses of Forest Breeding Birds through Broad Scale Analyses
Source: PLoS One. 2013 Feb 7;8(2):e55996. doi: 10.1371/journal.pone.0055996 (PMC3567043; doi:10.1371/journal.pone.0055996)
Supplement: Table S1 — Comparisons between logistic (non-threshold) and segmented (threshold) regression models for 24 study species. Red-eyed Vireo (Vireo olivaceus) was also included in the analysis but excluded from this table because none of the models converged. This species thus did not support a logistic or a threshold relationship with forest cover. In fact, it was present in all surveyed atlas blocks. We compared the AIC for all models using delta AIC (Δi), and selected a model when Δi >2 compared to the other model. We selected the model with the least number of parameters (non-threshold) when the difference between two models was Δi ≤2. In addition, we present the estimated threshold in the percentage of forest cover (%) and the associated standard error (SE) for the threshold models. For the selected models, we also present the Area Under the Curve statistic (AUC). The best model is highlighted in bold. (DOCX) [file pone.0055996.s003.docx]

**Table S1.**  Comparisons between logistic (non-threshold) and segmented (threshold) regression models for 24 study species. Red-eyed Vireo (*Vireo olivaceu*s) was also included in the analysis but excluded from this table because none of the models converged. This species thus did not support a logistic or a threshold relationship with forest cover. In fact, it was present in all surveyed atlas blocks. We compared the AIC for all models using delta AIC (∆_i_), and selected a model when ∆_i_ > 2 compared to the other model. We selected the model with the least number of parameters (non-threshold) when the difference between two models was ∆_i_ ≤ 2. In addition, we present the estimated threshold in the percentage of forest cover (%) and the associated standard error (SE) for the threshold models. For the selected models, we also present the Area Under the Curve statistic (AUC). The best model is highlighted in bold.

| **Species** | **Dynamic** | **Model** | **AIC** | **∆_i_** | % | **SE** | **AUC** |
| --- | --- | --- | --- | --- | --- | --- | --- |
| **Pileated Woodpecker** (*Dryocopus pileatus*) | Persistence | Threshold | 213.30 | 0.00 | 18.92 | 107.20 |  |
|  |  | **Non-Threshold** | **215.20** | **1.90** |  |  | **0.48** |
|  | Extinction | Threshold | 98.88 | 0.00 | 37.51 | 556.90 |  |
|  |  | **Non-Threshold** | **101.50** | **2.62** |  |  | **0.56** |
| **Yellow-bellied Sapsucker** (*Sphyrapicus varius*) | Persistence | **Threshold** | **113.21** | 0.00 | **34.61** | **10.97** | **0.84** |
|  |  | Non-Threshold | 116.10 | 2.89 |  |  |  |
|  | Extinction | Threshold | 31.92 | 0.00 | 42.65 | 142.60 |  |
|  |  | **Non-Threshold** | **33.65** | **1.73** |  |  | **0.65** |
| **Least Flycatcher** (*Empidonax minimus*) | Persistence | Threshold | 99.00 | 0.00 | 19.14 | 216.00 |  |
|  |  | **Non-Threshold** | **99.63** | **0.63** |  |  | **0.62** |
|  | Extinction | Threshold | 62.90 | 0.81 | 62.39 | 20.17 |  |
|  |  | **Non-Threshold** | **62.09** | **0.00** |  |  | **0.62** |
| **Common Raven** (*Corvus corax*) | Persistence | Threshold | 213.67 | 0.00 | 11.78 | 256.40 |  |
|  |  | **Non-Threshold** | **217.90** | **4.23** |  |  | **0.71** |
|  | Extinction | Threshold | 22.41 | 0.00 | 58.40 | 246.40 |  |
|  |  | **Non-Threshold** | **25.62** | **3.21** |  |  | **0.74** |
| **Black-capped Chickadee** (*Poecile atricapilla*) | Persistence | Threshold | NA |  |  |  |  |
|  |  | **Non-Threshold** | **26.53** | **0.00** |  |  | **0.91** |
|  | Extinction | Threshold | 13.48 | 0.00 | 89.39 | 161.00 |  |
|  |  | **Non-Threshold** | **15.49** | **2.01** |  |  | **0.76** |
| **Red-breasted Nuthatch** (*Sitta canadensis*) | Persistence | Threshold | 175.36 | 0.00 | 87.70 | 5.46 |  |
|  |  | **Non-Threshold** | **177.20** | **1.84** |  |  | **0.74** |
|  | Extinction | Threshold | 66.32 | 0.00 | 24.36 | 94.03 |  |
|  |  | **Non-Threshold** | **70.78** | **4.46** |  |  | **0.74** |
| **Brown Creeper** (*Certhia americana*) | Persistence | Threshold | 230.60 | 0.00 | 33.27 | 14.61 |  |
|  |  | **Non-Threshold** | **232.40** | **1.80** |  |  | **0.64** |
|  | Extinction | Threshold | 118.20 | 0.70 | 13.94 | 232.80 |  |
|  |  | **Non-Threshold** | **117.50** | **0.00** |  |  | **0.57** |
| **Winter Wren** (*Troglodytes troglodytes*) | Persistence | Threshold | 152.64 | 0.00 | 87.43 | 3.11 |  |
|  |  | **Non-Threshold** | **155.30** | **2.66** |  |  | **0.85** |
|  | Extinction | Threshold | 55.00 | 0.00 | 20.54 | 66.67 |  |
|  |  | **Non-Threshold** | **57.14** | **2.14** |  |  | **0.74** |
| **Hermit Thrush** (*Catharus guttatus*) | Persistence | Threshold | NA |  |  |  |  |
|  |  | **Non-Threshold** | **116.50** | **0.00** |  |  | **0.83** |
|  | Extinction | Threshold | 44.72 | 0.00 | 17.27 | 111.00 |  |
|  |  | **Non-Threshold** | **44.74** | **0.02** |  |  | **0.74** |
| **Veery (***Catharus fuscescens***)** | Persistence | Threshold | 53.33 | 0.72 | 87.40 | 11.51 |  |
|  |  | **Non-Threshold** | **52.61** | **0.00** |  |  | **0.78** |
|  | Extinction | Threshold | 39.16 | 0.42 | 86.18 | 12.16 |  |
|  |  | **Non-Threshold** | **38.74** | **0.00** |  |  | **0.69** |
| **Golden Crowned-Kinglet** (*Regulus satrapa*) | Persistence | Threshold | 202.61 | 0.19 | 96.65 | 2.26 |  |
|  |  | **Non-Threshold** | **202.80** | **0.00** |  |  | **0.73** |
|  | Extinction | **Threshold** | **159.11** | **0.00** | **75.27** | **7.00** | **0.62** |
|  |  | Non-Threshold | 161.60 | 2.49 |  |  |  |
| **Blue-headed Vireo** (*Vireo solitarius*) | Persistence | Threshold | 165.47 | 0.00 | 91.23 | 8.59 |  |
|  |  | **Non-Threshold** | **169.80** | **4.33** |  |  | **0.82** |
|  | Extinction | **Threshold** | **77.17** | **0.00** | **50.82** | **5.03** | **0.70** |
|  |  | Non-Threshold | 80.64 | 3.47 |  |  |  |
| **Black-and-white Warbler** (*Mniotilta varia*) | Persistence | **Threshold** | **75.77** | **0.00** | **87.96** | **5.83** | **0.82** |
|  |  | Non-Threshold | 79.52 | 3.75 |  |  |  |
|  | Extinction | **Threshold** | **39.70** | **0.00** | **91.20** | **5.67** | **0.78** |
|  |  | Non-Threshold | 41.90 | 2.20 |  |  |  |
| **Nashville Warbler** (*Vermivora ruficapilla*) | Persistence | Threshold | 220.71 | 0.00 | 98.27 | 0.46 |  |
|  |  | **Non-Threshold** | **222.20** | **1.49** |  |  | **0.68** |
|  | Extinction | Threshold | 153.17 | 0.00 | 87.37 | 5.59 |  |
|  |  | **Non-Threshold** | **153.50** | **0.33** |  |  | **0.53** |
| **Magnolia Warbler** *(Dendroica magnolia*) | Persistence | Threshold | 201.69 | 0.00 | 37.35 | 16.29 |  |
|  |  | **Non-Threshold** | **203.60** | **1.91** |  |  | **0.76** |
|  | Extinction | **Threshold** | **160.30** | **0.00** | **83.91** | **4.64** | **0.63** |
|  |  | Non-Threshold | 164.00 | 3.70 |  |  |  |
| **Black-throated B. Warbler** (*Dendroica caerulescens*) | Persistence | Threshold | 132.38 | 0.00 | 87.40 | 48.07 |  |
|  |  | **Non-Threshold** | **137.30** | **4.92** |  |  | **0.83** |
|  | Extinction | Threshold | 69.76 | 0.00 | 74.19 | 10.55 |  |
|  |  | **Non-Threshold** | **69.96** | **0.20** |  |  | **0.74** |
| **Yellow-rumped Warbler** *(Dendroica coronata*) | Persistence | **Threshold** | **151.47** | **0.00** | **19.45** | **3.83** | **0.80** |
|  |  | Non-Threshold | 161.00 | 9.53 |  |  |  |
|  | Extinction | **Threshold** | **112.03** | **0.00** | **78.67** | **3.76** | **0.73** |
|  |  | Non-Threshold | 118.50 | 6.47 |  |  |  |
| **Black-throated G. Warbler** (*Dendroica virens*) | Persistence | Threshold | 112.26 | 0.00 | 85.00 | 174.50 |  |
|  |  | **Non-Threshold** | **114.80** | **2.54** |  |  | **0.89** |
|  | Extinction | **Threshold** | **40.12** | **0.00** | **73.82** | **21.49** | **0.89** |
|  |  | Non-Threshold | 46.60 | 6.48 |  |  |  |
| **Blackburnian Warbler** (*Dendroica fusca*) | Persistence | **Threshold** | **124.83** | **0.00** | **56.26** | **6.61** | **0.90** |
|  |  | Non-Threshold | 135.10 | 10.27 |  |  |  |
|  | Extinction | **Threshold** | **77.98** | **0.00** | **69.91** | **5.26** | **0.78** |
|  |  | Non-Threshold | 84.01 | 6.03 |  |  |  |
| **Ovenbird** (*Seiurus aurocapilla*) | Persistence | Threshold | 22.90 | 0.00 | 42.41 | 656.10 |  |
|  |  | **Non-Threshold** | **23.83** | **0.93** |  |  | **0.95** |
|  | Extinction | Threshold | 12.40 | 0.00 | 37.26 | 34.29 |  |
|  |  | **Non-Threshold** | **14.74** | **2.34** |  |  | **0.90** |
| **Canada Warbler** (*Wilsonia canadensis*) | Persistence | Threshold | 213.97 | 0.00 | 92.53 | 3.33 |  |
|  |  | **Non-Threshold** | **215.60** | **1.63** |  |  | **0.73** |
|  | Extinction | **Threshold** | **211.70** | **0.00** | **87.40** | **3.55** | **0.61** |
|  |  | Non-Threshold | 216.70 | 5.00 |  |  |  |
| **Scarlet Tanager** (*Piranga olivacea*) | Persistence | Threshold | 132.46 | 0.00 | 16.08 | 58.71 |  |
|  |  | **Non-Threshold** | **133.00** | **0.54** |  |  | **0.62** |
|  | Extinction | Threshold | 84.80 | 0.00 | 21.21 | 50.46 |  |
|  |  | **Non-Threshold** | **85.29** | **0.49** |  |  | **0.61** |
| **Dark-eyed Junco** (*Junco hyemalis (hyemalis)*) | Persistence | **Threshold** | **148.97** | **0.00** | **57.12** | **7.43** | **0.86** |
|  |  | Non-Threshold | 156.60 | 7.63 |  |  |  |
|  | Extinction | **Threshold** | **85.01** | **0.00** | **58.65** | **5.90** | **0.72** |
|  |  | Non-Threshold | 91.32 | 6.31 |  |  |  |
| **White-throated Sparrow** (*Zonotrichia albicollis*) | Persistence | Threshold | 88.84 | 1.29 | 92.11 | 10.63 |  |
|  |  | **Non-Threshold** | **87.55** | **0.00** |  |  | **0.80** |
|  | Extinction | Threshold | 83.83 | 1.28 | 92.13 | 10.53 |  |
|  |  | **Non-Threshold** | **82.55** | **0.00** |  |  | **0.77** |
